# Antibiotic use in gastrointestinal surgery patients at a Vietnamese national hospital

**DOI:** 10.1186/s12876-022-02587-1

**Published:** 2022-11-22

**Authors:** Huyen Thi Nguyen, Quynh Thi Huong Bui, Tam Van Vo, Hien Thi Thu Pham, Thong Duy Vo

**Affiliations:** 1 Department of Clinical Pharmacy, Faculty of Pharmacy, University of Medicine and Pharmacy at Ho Chi Minh City, Ho Chi Minh City, Vietnam; 2 Department of Pharmacy, Thong Nhat Hospital, Ho Chi Minh City, Vietnam; 3 Faculty of Nursing and Medical Technology, University of Medicine and Pharmacy at Ho Chi Minh City, Ho Chi Minh City, Vietnam; 4 Department of Internal Medicine, Faculty of Medicine, University of Medicine and Pharmacy at City, 217 Hong Bang, Ward 11, Dis. 5, Ho Chi Minh City, 72714 Vietnam; 5 Department of Gastroenterology and Hepatology, Cho Ray Hospital, Ho Chi Minh City, Vietnam

**Keywords:** Prophylactic antibiotics, Invasive gastrointestinal surgery, Postoperative antibiotics

## Abstract

**Background:**

Invasive gastrointestinal surgery may be performed as an open or endoscopic procedure, such as laparoscopic semi-colon surgery, laparoscopic appendectomy, laparoscopic gastrectomy, and anal surgery, among other such operations. Regardless of the approach, the operative procedure interferes with the patient’s gastrointestinal tract, necessitating the rational use of prophylactic antibiotics to improve treatment outcomes and minimize postoperative infections.

**Objective:**

To investigate the prophylactic and postoperative antibiotic usage in patients who underwent invasive gastrointestinal surgery, and to identify factors associated with postoperative infection.

**Design:**

This descriptive, cross-sectional study included 112 patients who underwent invasive gastrointestinal surgery at the Department of Gastroenterology, Thong Nhat Hospital. We conducted a cross-sectional study in all inpatients aged 18 years and older, who underwent invasive gastrointestinal surgery between January 2020 and December 2020. We recorded patient characteristics, the administration and appropriateness of antibiotics, as well as treatment outcomes. The appropriateness of prophylactic and postoperative antibiotic usage was assessed based on 2015 Vietnamese national guideline for antibiotic use. Multivariable logistic regression analysis was used to determine the factors associated with postoperative infection.

**Results:**

Patients’ mean age was 59.7 ± 17.2 years. Most surgeries (89.3%) were clean-contaminated procedures. The rates of appropriate types of antibiotics selected, doses, and overall rates of appropriateness of antibiotic prophylaxis were 68.0%, 76.4% and 54.7%, respectively. Of the patients investigated, 34.8% had at least one sign of postoperative infection; the overall appropriate rate of postoperative antibiotic was 38.5%. Old age was associated with postoperative infection and longer length of hospitalization.

**Conclusion:**

Implementation of the guidelines recommended for the prophylactic and therapeutic use of antibiotics is essential to improve treatment outcomes.

## Introduction

Invasive gastrointestinal surgery may be performed as an open or laparoscopic procedure, such as colectomy, appendectomy, small bowel resection, gastrectomy, and anal surgery. Regardless of the surgical approach, operative procedures involving the gastrointestinal tract can cause serious complications, such as anastomotic leakage and life-threatening infections [1].

Comprehensive treatment and care, including maintenance of the nutritional status and safe and effective use of drugs are important in patients who undergo invasive gastrointestinal surgery. Treatment outcome is affected by age, comorbidities, surgical complexity, quality of preoperative care, and optimal management of postoperative recovery [2]. Infection, wound pain, and malnutrition are the most common clinical conditions observed in patients postoperatively.

Advances in technology have led to the introduction of novel and sophisticated techniques and the availability of superior levels of clinical care; therefore, the effectiveness of gastrointestinal surgery has significantly improved over the past few decades. Currently, postoperative infections are the most common risk factors associated with hospital readmission and increased cost of treatment [3], and surgical site infections are the most common adverse events and an important cause of mortality in surgical patients worldwide. Surgical site infections represent a common type of nosocomial infection (38%) and are estimated to increase by 2–5%/year in patients who undergo surgical procedures each year [4]. Surgical site infection rates are significantly high in patients who undergo gastrointestinal surgery owing to colonization by gut microbiota [5]. Based on data shared by the American Healthcare Network, surgical site infection rates are highest after colon and rectal surgery (9.47% and 26.67%, respectively). To date, the lowest surgical site infection rate was 3.47% observed in patients who underwent appendectomy [6].

Adequate precautions including surgical hand disinfection, optimal blood glucose control, and strict adherence to aseptic procedures in the operating room and during wound care are among the measures that can avoid surgical site infections in approximately 40–60% patients [7]. Prophylaxis with antibiotics is also one of the measures to reduce surgical site infections [8]. The timely and rational use of prophylactic antibiotics in clean and clean-contaminated surgery is the most important precaution that can effectively minimize surgical site infection rates [4, 9]. Postoperative infections are associated with prolonged hospitalization [10], increased treatment costs, and even mortality [11].

We conducted this study to investigate the prophylactic and postoperative antibiotic usage in patients who underwent invasive gastrointestinal surgery, and to identify factors associated with postoperative infection.

## Materials and methods

### Study design

We conducted a descriptive cross-sectional study in all inpatients aged 18 years and older, who underwent invasive gastrointestinal surgery between January 2020 and December 2020 at Thong Nhat Hospital, a National Hospital in South Vietnam. Patients who underwent more than one surgery during hospitalization were excluded from the study. We reviewed patients’ medical records and analyzed the following data: patient characteristics, antimicrobial therapy, and treatment outcomes including postoperative infections and length of hospitalization.

### Data collection

Data for the patients were recorded using a data collection form for each study subject. This form was used to collect the basic data, including age, gender, initial estimated glomerular filtration rate (eGFR, mL/min/1.73 m^2^), comorbidities. We also collected data pertaining to surgical procedures such as indication, surgical procedure, surgical classification. In addition, we recorded data relating to preoperative antibiotic used (indication, selection, dose, timing of the initial administration, and intraoperative re-dosing and duration) and postoperative ones (selection and dose).

### Definitions

Surgical classification was categorized into four groups—clean, clean-contaminated, contaminated, and dirty/infected. A clean surgery is defined as an incision in which no infection, inflammation and sterile error is encountered, and during which the respiratory, alimentary, and genitourinary tracts are not entered. A clean-contaminated surgery is an incision that entered the respiratory, alimentary or genitourinary tract under controlled conditions and encountered no contamination. A contaminated surgery is an incision undertaken during an operation in which there is a major break in sterile technique or gross spillage from the gastrointestinal tract, or an incision in which acute, non-purulent inflammation is encountered. Open traumatic wounds that are more than 12–24 h old also fall into this category. Dirty or infected surgery is an incision undertaken during an operation in which the viscera are perforated or when acute inflammation with pus is encountered during the operation (for example, emergency surgery for fecal peritonitis), and for traumatic wounds where treatment is delayed, and there is fecal contamination or devitalized tissue present [12, 13].

The sign of postoperative infections included purulent discharge from the incision site, positive results on microbiological culture indicated by discharge of fluid from a closed incision, surgery at sites with at least one clinical manifestation of inflammation (such as swelling, heat, redness, pain), positive culture results, or an infection diagnosed by the surgeon based on the Center of Disease Control (CDC) criteria [13].

The treatment outcomes were the rate of postoperative infections and the length of hospitalization.

We evaluated the appropriateness of prophylactic antibiotic therapy in patients with clean or clean- clean-contaminated surgery with regard to the choice of antibiotic (antibiotic indication), type, dosage, timing, additional dosage, and duration of administration. Criteria for appropriateness of antibiotic administration included physician adherence to guideline-recommended antimicrobials from the 2015 Vietnamese national guideline [14]. We evaluated the appropriateness of postoperative antibiotic treatment with regard to the type and dosage of administration in all patients. The criterion for the appropriateness of antibiotics was physician adherence to antimicrobial usage guidelines from the 2015 Vietnamese national guideline of antibiotic usage [14]. Postoperative antibiotic usage was defined as the appropriate type and dosage of antibiotic.

### Statistical analysis

Sample characteristics were described using the median and interquartile range (IQR) for continuous variables that showed a normal distribution, the mean and standard deviation (SD) for other continuous variables, and percentages for categorical variables. All data were analyzed using the SPSS software, version 20.0. Data are expressed as mean ± SD, median (IQR [25th to 75th percentile]) or percentages. Multivariable logistic regression analysis was used to determine the factors, such as age, sex, number of comorbidities and rationale of antibiotic prophylaxis, associated with the postoperative infection rate. Multivariate linear regression analysis was used to identify factors such as age, sex, surgical classification, number of comorbidities, postoperative infection, and rationale for antibiotic prophylaxis and therapy, associated with the length of hospitalization. *P* value < 0.05 was considered statistically significant.

## Results

### General characteristics of the study patients

Patients’ mean age was 59.7 ± 17.2 years (62.5% were men). Most patients had stable renal function with an estimated glomerular filtration rate > 60 mL/min/1.73 m^2^ (86.6%). Patients were mainly hospitalized for cancer (42.0%), and laparoscopic surgery was the procedure indicated in most patients, with laparoscopic colectomy being the predominant operation (35.7%). Most patients underwent clean or clean-contaminated surgery (94.7%) (Table 1).

**Table 1:** General characteristics of the study population

Characteristic	Frequency	Percentage (%)
*Age*		
Mean ± SD	59.7 ± 17.2	
≥ 65 years	42	37.5
< 65 years	70	62.5
*Gender*		
Men	73	65.2
Women	39	34.8
*Initial eGFR*		
≥ 60 mL/min/1.73 m^2^	97	86.6
< 60 mL/min/1.73 m^2^	15	13.4
No comorbidities	52	46.4
*Number of comorbidities*		
1 Comorbidity	38	33.9
≥ 2 Comorbidities	22	19.6
*Type of comorbidity*		
Hypertension	27	23.1
Type 2 diabetes	13	11.6
Gastritis	8	7.1
Stomach ulcers	5	4.5
Dyslipidemia	4	3.6
Other comorbidities^d^	12	10.7
*Surgical indication*		
Cancer	47	42.0
Peritonitis	31	27.7
Bowel obstruction	20	17.9
Others^a^	14	12.5
*Surgical procedure*		
Laparoscopic resection/semi-transverse/sigmoid surgery	40	35.7
Laparoscopic appendectomy/cholecystectomy	14	12.5
Laparoscopic/semi-gastric surgery	13	11.6
Laparoscopic surgery to suture a perforation	4	3.6
Abscess removal surgery	5	4.5
Laparoscopic small bowel surgery	4	3.6
Laparoscopic surgery to open/close the colostomy	8	7.1
Others^c^	24	21.4
*Surgical classification*		
Clean	6	5.4
Clean-contaminated	100	89.3
Contaminated	6	5.4

eGFR: Estimated glomerular filtration rate, SD: standard deviation

### Prophylactic antibiotics

We assessed the appropriateness of prophylactic antibiotics in 106 patients, who underwent clean or clean- clean-contaminated surgery (Table 2). The appropriateness of prophylactic antibiotic indication in our study was 97.2%. Cefoxitin (64.2%) and metronidazole (21.7%) were the most common antibiotics administered for prophylaxis in this study. No patient had a history of allergy to β-lactam antibiotics. Most of the 97 patients who were prescribed prophylactic antibiotics received monotherapy (76.3%). Cefoxitin is the most commonly used as monotherapy antibiotic (49.5%). The percentage of antibiotics dosage that differed from the recommended dosage is 23.6%. We observed that 100% prophylactic antibiotics were used within 30–60 min before creation of the skin incision.

**Table 2:** Distribution of prophylactic antibiotics administered in this study

Pharmacological group	Antibiotics	Quantity	Percentage (%)
No prophylactic antibiotics administered		9	8.5
β-lactam antibiotics (n = 87)	Cefoxitin	68	64.2
	Ceftriaxone	9	8.5
	Cefazolin	9	8.5
	Cefoperazone/sulbactam	1	0.9
Fluoroquinolones (n = 10)	Moxifloxacin	9	8.5
	Ciprofloxacin	1	0.9
5-nitro-imidazoles (n = 23)	Metronidazole	23	21.7

Notably, no patient received any intraoperative antibiotic. The mean surgical time was 116.5 ± 40.5 min, and no severe intraoperative blood loss (> 1500 mL) was observed. The duration of surgery did not exceed the time required to repeat the dose of the prophylactic antibiotic selected for each patient. Prophylactic antibiotic administration was continued, despite the lack of any sign of infection, over 24 h postoperatively in 12.4% of patients. Of the 106 patients who underwent clean and clean-contaminated surgery, antibiotic administration was justifiably indicated in 103 patients, among whom 6 patients from the clean surgery group did not require prophylactic antibiotic administration, and 97 patients from the clean-contaminated surgery group used only one prophylactic antibiotic. The overall appropriate rate of prophylactic antibiotic was 54.7% (Table 3).

**Table 3:** Rate of appropriateness of prophylactic antibiotic in this study

Serial No	Level of appropriateness	Frequency	Percentage (%)
1	Indications (n = 106)	103	97.2
2	Type (n = 103)	66	68.0
3	Dosage (n = 97)	74	76.4
4	Timing (n = 97)	97	100
5	Additional dose (n = 97)	97	100
6	Duration of administration (n = 97)	85	87.6
7	Overall appropriateness (n = 106)	58	54.7

### Postoperative antibiotic use

The median duration of postoperative antibiotic use was 9 (7–12) days; the most prolonged duration of antibiotic use was 28 days. β-lactam and fluoroquinolone were the most common antibiotic groups prescribed (Table 4). The overall rate of appropriateness of antibiotic treatment was 38.5%.

**Table 4:** Pharmacological classification of antibiotics administered postoperatively

Pharmacological group	Antibiotics	Quantity	Prescriptions (%)
β-lactam antibiotics (n = 39)	Cefoxitin	8	20.5
	Imipenem/cilastatin	14	35.9
	Ceftriaxone	11	28.2
	Ertapenem	5	12.8
	Cefoperazone/sulbactam	1	2.7
Fluoroquinolones (n = 19)	Moxifloxacin	18	46.2
	Ciprofloxacin	1	2.7
Aminoglycosides (n = 8)	Amikacin	8	20.5
5-nitro-imidazoles (n = 16)	Metronidazole	16	41.0

### Treatment outcomes

In this study, 39 patients (34.8%) developed at least one sign of infection; 14 patients were diagnosed with superficial surgical site infection and 25 patients with one sign of surgical site infection. The median length of hospitalization was 14 (10–20) days (maximum duration 36 days). Our results show that old age was associated with a high risk of postoperative infection and longer length of hospitalization (Tables 5 and 6).

**Table 5:** Factors associated with surgical site infection

	OR	95% Confidence interval		*p* value
		Lower	Upper	
Age	1.036	1.010	1.063	**0.007**
Sex (women)	1.040	1.011	1.071	0.368
*Surgical classification*				
Clean	–	–	–	–
Clean-contaminated	3.029	0.280	0.362	0.362
Contaminated	1.251	0.054	0.889	0.889
Overall appropriateness of administration of prophylactic antibiotics (yes)	0.642	0.271	1.522	0.315

OR: Odds ratio

**Table 6:** Factors associated with the length of postoperative hospitalization

	Beta	95% Confidence Interval		*p* value
		Lower	Upper	
Age	0.157	0.084	0.230	**< 0.001**
Sex	0.790	− 1.566	3.145	0.508
Number of comorbidities	− 0.393	− 1.636	0.850	0.532
Surgical site infection	− 1.983	− 4.551	0.586	0.129
Overall appropriateness of prophylactic antibiotic administration	0.112	− 0.150	0.373	0.398
Overall appropriateness of antibiotic treatment	− 0.249	− 0.516	0.018	0.068

## Discussion

In our study, more than 33% of patients > 65 years old. Advanced age is a risk factor for postoperative infection and malnutrition [15–19]. Most surgeries in the study were classified as clean-contaminated (89.3%) procedures. According to Altemeier's surgical wound classification based on the risk of surgical infection, the risk of postoperative infection was as follows: 1–5% (clean incisions), 5–10% (clean-contaminated incisions) [20]. A cohort study showed that the rate of postoperative infection was 5.9% in cases of clean open surgery and 46.4% and 3.2% in open and laparoscopic clean-contaminated surgery, respectively [16].

### Prophylactic antibiotics

The appropriateness of prophylactic antibiotic indication in our study was 97.2%**,** which is higher than that reported by Malavaud et al. [17], Durando et al. [18], and Pittalis et al. [19], who observed rates of 88.1%, 70.3% and 95%, respectively. This finding may be attributable to the increasing prevalence of nosocomial infections and drug resistance in Vietnam and the greater attention to postoperative infectious complications and the use of preoperative antibiotic therapy. Most patients in our study received prophylactic cefoxitin (64.2%) and metronidazole (21.7%). Cefoxitin is a 2nd generation broad-spectrum cephalosporin with a wide range of anaerobic coverage and is therefore commonly indicated for prophylaxis in gastrointestinal procedures, such as cholecystectomy, colorectal surgery, and operations for small bowel obstruction [21, 22]. Metronidazole and cefazolin combination therapy is recommended for operations with a high risk of anaerobic bacterial contamination, such as biliary tract surgery, laparoscopy, laparoscopic appendectomy, operations for small bowel obstruction, and colon surgery [23]. Statistical evidence suggests that cefoxitin and metronidazole constitute a high percentage of prophylactic antibiotics administered for gastrointestinal surgery. Most patients were prescribed a single prophylactic antibiotic (76.3%). Commonly used prophylactic antibiotics include cefoxitin (49.5%), ceftriaxone (6.2%), and cefazolin (9.3%).

We observed that the choice of antibiotics used was appropriate in 68.6% of patients in this study. This rate is lower than that reported by Malavaud et al. [17] (91.9%) and Pittalis et al. [19] (84.5%), which is attributable to the differences in hospital practices and policies adopted in our study and those followed by the hospitals included in the aforementioned studies. In fact, prophylactic combination therapy using cefoxitin and metronidazole is widely used during gastrointestinal surgery for effective coverage against anaerobic bacteria, although this combination is not reasonable [24–26]. A systematic review of 18 studies that investigated adherence to guidelines for antibiotic prophylaxis observed that the rates of appropriateness of antibiotic prophylaxis ranged from 22 to 95% [27].

We observed that prophylactic antibiotic usage was 100% within 30–60 min before creation of the skin incision. Current guidelines recommend that prophylactic antibiotics should be administered within 60 min preoperatively and close to the time of creation of the skin incision (120 min for vancomycin and fluoroquinolones) [28, 29]. The timing of administration is an important determinant of successful antibiotic prophylaxis. Microbes that cause postoperative infection primarily invade the body during surgery (between the time of creation of the skin incision and wound closure) [28]. In a study performed by Koch et al. [22], the postoperative infection rate was lower in those who received prophylactic antibiotics within 60 min before the skin incision than in those who received prophylactic antibiotics later than 60 min before the skin incision. Several studies have reported that the effectiveness of antibiotic prophylaxis is maximum when administered during the 60-min period before creation of the skin incision [23, 24]. A study by Weber et al. [23] showed that the postoperative infection rate was higher in those who received prophylactic antibiotics between 0 and 30 min before the skin incision than in those who received prophylactic antibiotics over approximately 30–60 min prior to creation of the skin incision. In contrast, Steinberg et al. [24] observed no difference in the postoperative infection rate in patients who received prophylactic antibiotics within 0–30 min or 30–60 min.

No patient in our study received additional doses intraoperatively. Based on the clinical practice guidelines for antimicrobial prophylaxis in surgery, patients in whom the surgical time is > 2 times the T_1/2_ of the drug and those with intraoperative blood loss > 1500 mL in adults tend to require additional antibiotic prophylaxis to ensure adequate blood and tissue antibiotic concentrations [4, 20]. In our study, we observed that the surgical time did not exceed 120 min in patients who received cefoxitin; therefore, the interval between repeat doses of cefoxitin was not exceeded. Moreover, repeat dose intervals of ceftriaxone (6 h), cefazolin (4 h), and metronidazole (6 h) were longer than the surgical time. Additionally, intraoperative blood loss > 1500 mL did not occur in any patient in our study (based on patients’ surgical record). Therefore, repeat doses of prophylactic antibiotics were not required. The rate of appropriate dose supplementation in this study was 100%, which was higher than the rates reported by Goede et al. [25] (54.9%).

Prophylactic antibiotic usage beyond 24 h after treatment completion was observed in 52.6% of patients, of which 12.4% patients continued treatment without a justifiable indication. A study performed across 9 provincial and central hospitals in 2009 reported that 94.6% of patients received prolonged postoperative prophylactic antibiotics [26], which may have been due to the notion prevalent among surgeons regarding the need for long-term administration of antibiotics to prevent postoperative infections. However, per recommendations, most routine surgeries usually require administration of only a prophylactic dose. Antibiotic prophylaxis is not required beyond 24 h postoperatively for most surgeries (or beyond 48 h for cardiac surgery) [20]. The appropriateness of the rate of prolonged antibiotic prophylaxis in our study was 87.6%, which was higher than that reported by Pittalis et al. [19] (48%). Gouvea et al. [21] showed that the reasonable rate of duration of antibiotic prophylaxis ranged from 5.8 to 91.4%.

The overall rate of appropriateness of antibiotic prophylaxis in our study was 54.7%, which is similar to that reported by Malavaud et al. [17] (58.3%). The similarity in results may be attributed to the fact that the types of surgeries performed and the criteria for criteria for antibiotic selection were nearly identical between our study and the study performed by Malavaud et al. [17].

### Postoperative antibiotic use

In this study, the rate of appropriate postoperative antibiotic type was 51.3%, and combination antibiotic therapy (dual anaerobic coverage therapy) resulted in inappropriate antibiotic treatment. The overall rate of appropriate antibiotic treatment was 38.5%. Culture or antibiogram were not performed in any patient in this study, and treatment was based exclusively on experience*.* This is one of the limitations associated with the use of antibiotics in the Department of Surgery, which should be addressed in future research. Adherence to recommended guidelines is important to reduce inappropriate antibiotic use and to improve treatment effectiveness, to benefit both patients and hospitals.

### Treatment outcomes

Surgical site infection is one of the most common infections associated with health care. Therefore, there are many studies to investigate postoperative infection, including surgical site infection after gastrointestinal surgery. In this study, 34.8% of patients had at least one sign of infection, which is higher than that reported by Bhangu et al. [30], Wang et al. [31], Alkaaki et al. [16], who reported rates of 2–5%, 5.2% and 16.3%, respectively. This difference can be explained as follow, we recorded cases of patients with at least one sign of infection, while other studies recorded the proportion of patients with accurate diagnosis of surgical site infection.

The median length of hospitalization was 14 (10–20) days, which is similar to that reported by Jakobson et al. [32] (14.5 ± 10 days) and higher than that reported by Alkaaki et al. [16], the median postoperative hospital stay was 2 days for patients without SSI, compared to 13 days for those with SSI.

We observed that increasing age increased the risk of postoperative, which is similar to findings reported by Neumayer et al. [27], who observed a higher risk of postoperative infection in patients aged over 40 years old than in those aged under 40 years. A study performed in the United States showed that the risk of postoperative infection increased 1.2-fold with every 1-year increase in age among patients aged above 65 years [28].

Furthermore, we observed that advanced age was associated with longer length of hospitalization; this result is similar to that reported by Chong et al. [29], who observed that advanced age was associated with the longer length of hospitalization in patients who underwent laparoscopic cholecystectomy.

Following are the limitations of this study: (a) This retrospective observational study was based on data obtained from patients’ medical records; therefore, we did not consider all potential confounders that may have affected our results. (b) Owing to the small sample size and single-center study design, our findings may not accurately reflect the effect of prophylactic and therapeutic antibiotics in patients who undergo gastrointestinal surgery. Further large-scale studies are warranted to gain a deeper understanding of this subject.

## Conclusions

The overall rates of appropriateness of antibiotic prophylaxis was 54.7%. The overall appropriate rate of postoperative antibiotic was 38.5%. Our study highlights that old age was a risk factor for postoperative infection and was associated with longer length of hospitalization. These findings suggest the importance of strict adherence to guidelines for the administration of prophylactic and therapeutic antibiotics, particularly in older patients, to improve treatment outcomes.

## Data Availability

The datasets used and/or analysed during the current study are available from the corresponding author on reasonable request.

## Abbreviations

IQR: Interquartile range
SD: Standard deviation
OR: Odds ratio
CI: Confidence interval
eGFR: Estimated glomerular filtration rate
